# Synthesis, crystal structure and thermal properties of *N*′-[(*E*)-3,5-di-*tert*-butyl-2-hy­droxy­benzyl­idene]-2-hy­droxy­benzohydrazide ethanol quatersolvate

**DOI:** 10.1107/S2056989025006504

**Published:** 2025-07-29

**Authors:** Mamadou Lo, Bineta Sene, Anastasie Manga, Aissatou Alioune Gaye, Abdoulaye Gassama, Amadou Diop, Arie van der Lee, Sébastien Richeter

**Affiliations:** a Département de Chimie, UFR des Sciences et Technologies, Laboratoire de Chimie Physique des Matériaux (LCPM), BP 523, Ziguinchor, Senegal; b LCCOB Département de Chimie, Faculté des Sciences et Techniques, Université Cheikh Anta Diop, Dakar, 10700, Senegal; c Research Development chez Delmar Chemical, 364, rue Juneau, H8R 3X8, Montréal, Québec, Canada; d Institut Européen des Membranes, Université de Montpellier, CNRS, ENSCM, 34095 Montpellier, France; e ICGM, Univ. Montpellier, CNRS, ENSCM, 34293 Montpellier, France; Universidade de Sâo Paulo, Brazil

**Keywords:** crystal structure, salicylic acid, acyl­hydrazone

## Abstract

In the structure of the title compound, 4C_22_H_28_N_2_O_3_·C_2_H_6_O, mol­ecules composed of an acyl­hydrazone segment bridging a 3,5-di-*tert*-butyl­phenol unit and a 2-hy­droxy­phenol unit form columnar quartet channels along the *a*-axis, within which disordered ethanol solvent mol­ecules reside in two inversion-centre-related positions. Inter­molecular hydrogen bonds assemble the channels between the columnar mol­ecules through the hosted ethanol mol­ecule, complemented by weaker van der Waals-type inter­actions. These weak van der Waals inter­actions also hold the columns together. Additionally, the title compound demonstrated notable thermal stability at temperatures of up to 251°C. The sharp melting point peak observed in the DSC confirmed its crystalline structure.

## Abstract 2

The structure of the title compound, 4C_22_H_28_N_2_O_3_·C_2_H_6_O, exhibits a one-dimensional pore structure composed of channels formed by mol­ecules having an acyl­hydrazone segment connecting a 3,5-di-*tert*-butyl­phenol unit and a 2-hy­droxy­phenol unit. The channels contain disordered ethanol mol­ecules.

## 1. Chemical context

2-Hy­droxy­benzoic acid, commonly known as salicylic acid, is a natural or synthetic chemical compound whose medicinal properties have been utilized for over 2000 years in the treatment of dermatological diseases (Arif, 2015 ▸). Furthermore, its synthetic derivatives have demonstrated efficacy in the pharmaceutical field, particularly as active ingredients in various medications (Bai *et al.*, 2020 ▸; Ekinci *et al.*, 2011 ▸). Among these, acetyl­salicylic acid, widely recognized by the trade name aspirin, is the most renowned and extensively used derivative in medicine owing to its analgesic, anti­pyretic, and non-steroidal anti-inflammatory properties (Montinari *et al.*, 2019 ▸). Acyl­hydrazone-type Schiff bases are obtained through a condensation reaction, either catalysed or non-catalysed, between an aldehyde and hydrazide, releasing water as a byproduct. The acyl­hydrazone structure, which combines an imine and carbonyl functional group, imparts significant pharmacological properties, making it a key pharmacophore in medicinal chemistry (Kassab, 2024 ▸). Acyl­hydrazones exhibit a wide range of biological activities (Socea *et al.*, 2022 ▸), which justifies their presence in several drugs (Thota *et al.*, 2018 ▸). Additionally, ligands featuring acyl­hydrazone moieties are extensively used in coordination chemistry because of their ability to form complexes with transition metals (Basaran *et al.*, 2024 ▸). These ligands have diverse applications, including the detection of metal ions by fluorescence sensing (Muthukumar *et al.*, 2020 ▸; Nandakumar *et al.*, 2025 ▸) and the synthesis of heterocycles in organic chemistry (Lv *et al.*, 2021 ▸). Moreover, 3,5-di-*tert*-butyl­benzaldehyde, when combined with hydrazides derived from hy­droxy­benzoic acid, yields acyl­hydrazones with remarkable potential as enzyme inhibitors (Maniak *et al.*, 2020 ▸; Ghatak *et al.*, 2014 ▸). In this context, we successfully isolated and characterized the crystallographic structure of the title compound, an acyl­hydrazone composed of 3,5-di-*tert*-butyl­phenol and 2-hy­droxy­phenol units. The coexistence of these two moieties within the same mol­ecule enhances its anti­oxidant properties and provides the title compound with promising multidentate ligand capabilities for transition-metal complexation.
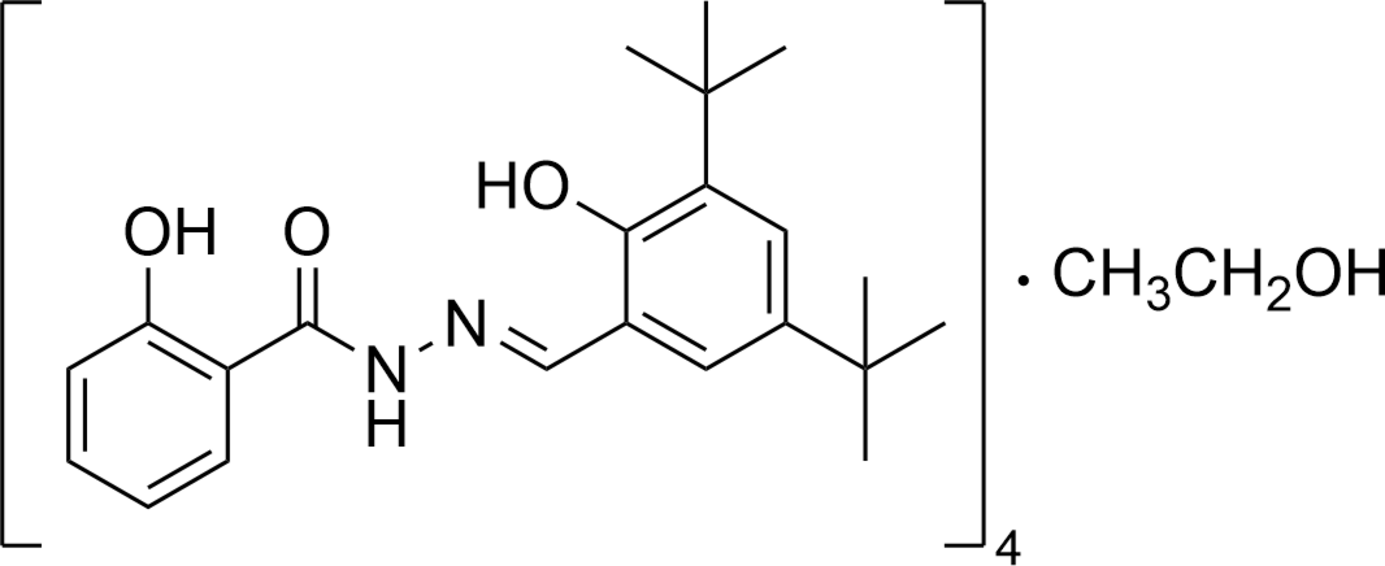


## 2. Structural commentary

**Figure 1:**
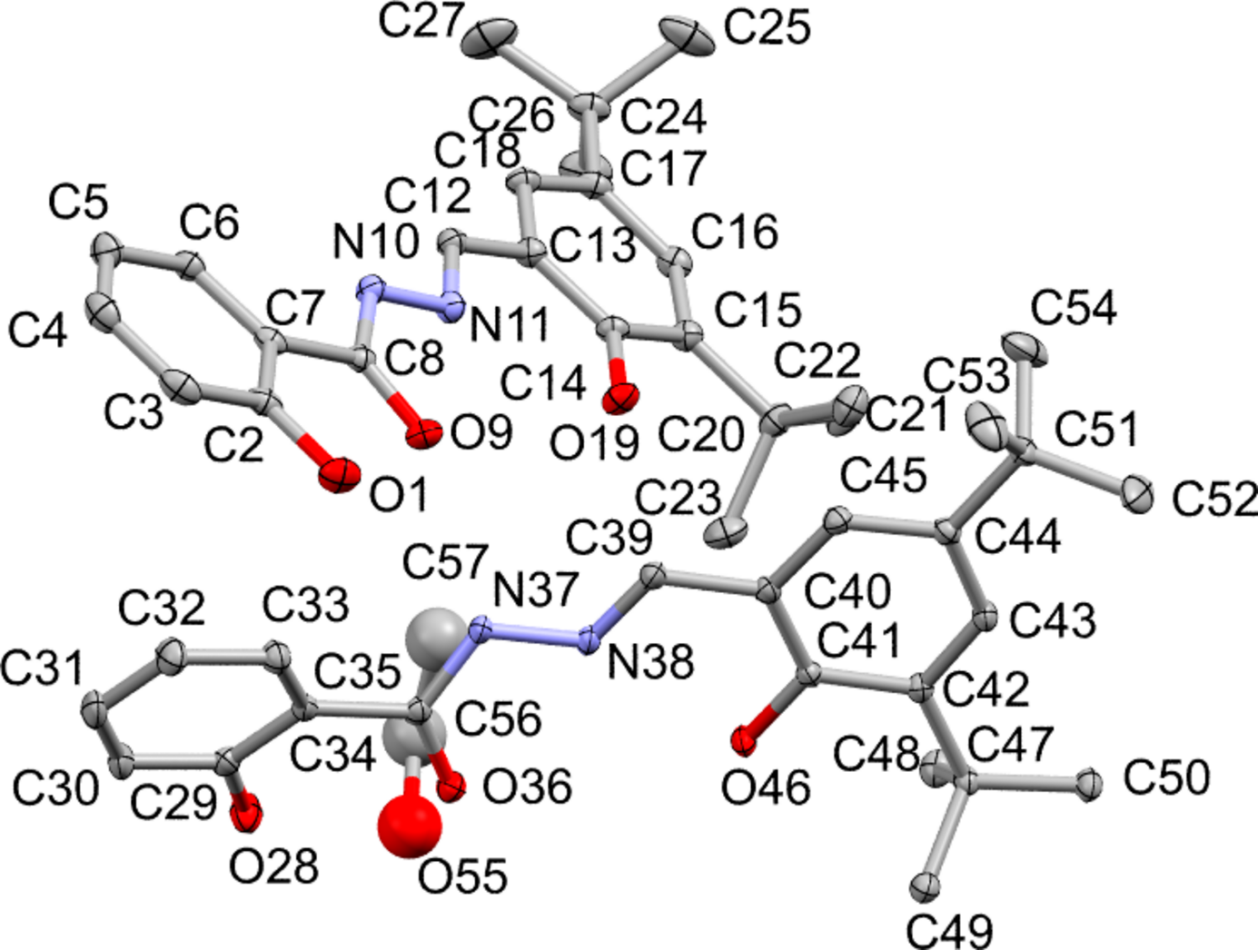
A view of the asymmetric unit of the title structure showing the atom-labelling scheme. The atomic displacement ellipsoids are drawn at the 30% probability level and hydrogen atoms have been omitted for clarity. The occupancy probability of the ethanol mol­ecule is 50%.

The asymmetric unit consists of two mol­ecules of the title compound and half of the ethanol solvent mol­ecule (Fig. 1 ▸). The latter mol­ecule is disordered across two distinct positions related by an inversion centre. Intra­molecular hydrogen bonds are found in both independent title mol­ecules, occurring between the hy­droxy group of the phenol ring and the carb­oxy group of the acyl­hydrazone linker, as well as between the hy­droxy group of the 3,5-di-*tert*-butyl­phenol group and the imine nitro­gen atom of the acyl­hydrazone linker. The r.m.s. deviation between the two independent mol­ecules of the title compound is 0.5234 Å (0.3766 Å with inversion), where the largest differences occur in the *tert*-butyl groups. A default *Mogul* check (Bruno *et al.*, 2004 ▸) yielded one unusual torsion angle: O36—C35—C34—C29, −26.1 (3)° for 2348 hits of related fragments with a local density value of only 2.8%. The local density value is the percentage of the observed database values that fall within 10° of the query value, here the torsion angle of this mol­ecule. The value of the corresponding torsion angle in the second independent title mol­ecule (O9—C8—C7—C2) is 8.6 (3)°, which falls within the accepted statistical range with a local density value of 12.6%. The ideal geometry of this torsion angle is planar, corresponding to a torsion angle of 180°. The largely deviating O36—C35—C34—C29 torsion angle is most probably caused by the presence of ethanol mol­ecules in the cavities, which attract the hy­droxy group of the phenol ring and rotate the phenol ring around the bond connecting it to the carb­oxy group.

## 3. Supra­molecular features

**Figure 2:**
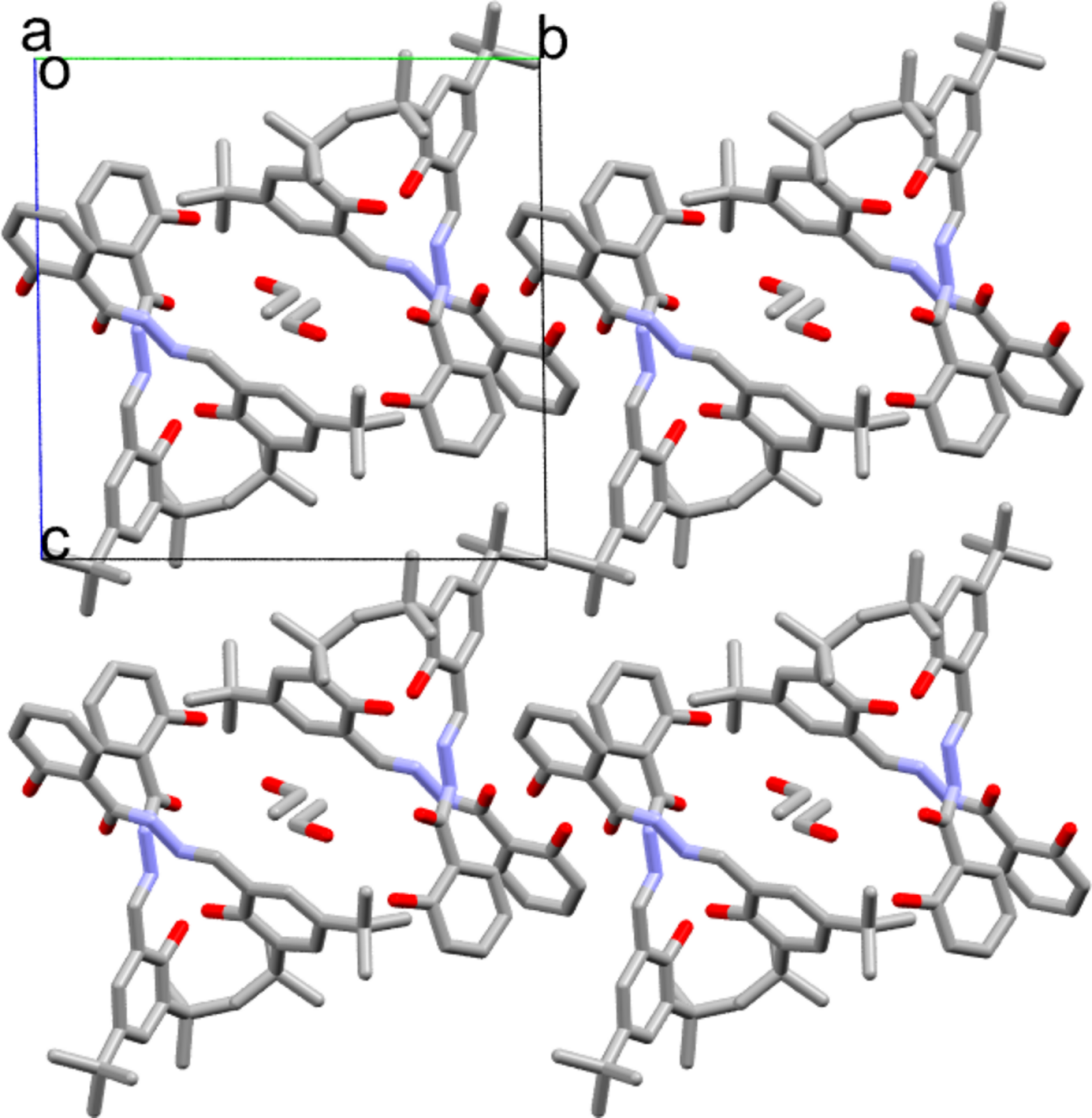
Projection of the structure of the title compound along the *a* axis. The two disordered parts of the ethanol mol­ecule are shown.

**Figure 3:**
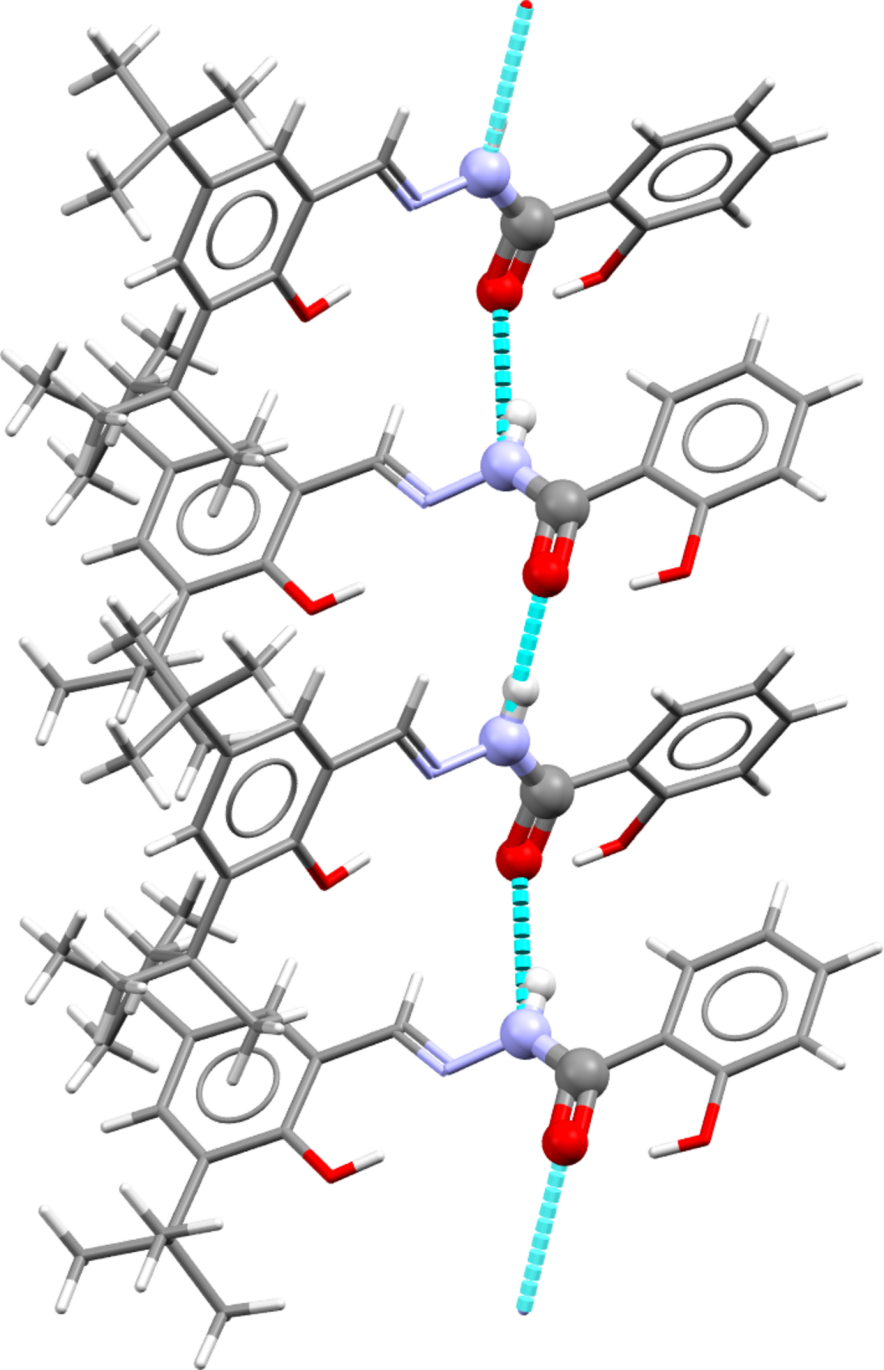
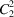
(8) chain in the structure of the title compound. Hydrogen-bond donor–acceptor inter­actions are indicated as light-blue dashed lines.

The ethanol mol­ecules are found in straight channels running parallel to the *a*-axis. Each channel consists of four title mol­ecules arranged in a quartet formation stacked along the channel axis (Fig. 2 ▸). The channels are stacked in the *b-* and *c*-axis directions. A slightly larger inter­molecular O1⋯N37 hydrogen-bond inter­action could align the channels in the *b*-axis direction [3.046 (2) Å, with the hypothetical atom in the correct position], but O1 is donated solely to the intra­molecular O9 atom. Therefore, no relatively strong hydrogen-bond inter­actions occur between neighbouring channel stacks in either the *b*- or *c*-axis direction. No ring inter­actions with a centroid-to-centroid distance below 2 Å are present, but two CH⋯centroid distances below 3 Å are indeed observed: C5—H5⋯*Cg*3(*x* − 1, *y*, *z*) (2.87 Å) and C27—H27*BCg*3(−*x*, −*y* + 1, −*z* + 1) (2.88 Å), where *Cg*3 is the ring formed by C29–C34. However, these weak inter­actions do not mediate between neighbouring channel stacks or between the mol­ecules constituting each quartet stack. The assembly of each stack is strengthened by inter­molecular hydrogen-bond inter­actions (Table 1 ▸), forming two infinite 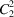
(8) chains along the *a*-axis direction, constituting each half of a channel wall (Fig. 3 ▸). The two halves of the channel walls are connected to each other by relatively weak van der Waals-type inter­actions.

An analysis of the coordination likelihoods of the inter­molecular hydrogen bonds, based on statistical models using version 5.46 version of the Cambridge Structural Database (Groom *et al.*, 2016 ▸; with November 2024 updates) and employing Alvarez’ bond radii (Alvarez, 2013 ▸), shows that all hydrogen bonds have the expected coordination for the first of the two parts in the structure. Exceptions include the aromatic hy­droxy group O28—H28, which is usually not an inter­molecular donor in that position; the O36 acceptor of the acyclic amide group, which is observed to have three inter­molecular donor groups, a very rare occurrence; and the O9 acceptor of the acyclic amide group of the other independent mol­ecule with a coordination number of one. In contrast, the likelihood of this type of acceptor having two donors is slightly lower than 0.5, whereas the possibility of having one donor is greater than 0.5. The observed hydrogen bonds in the other parts differ somewhat because O36 has only one donor, and the O28–H28 hy­droxy group has no acceptor, as expected. Conversely, the ethanol acceptor and donors do not have any inter­molecular hydrogen-bond donors or acceptors, which is unexpected.

## 4. Thermal properties

**Figure 4:**
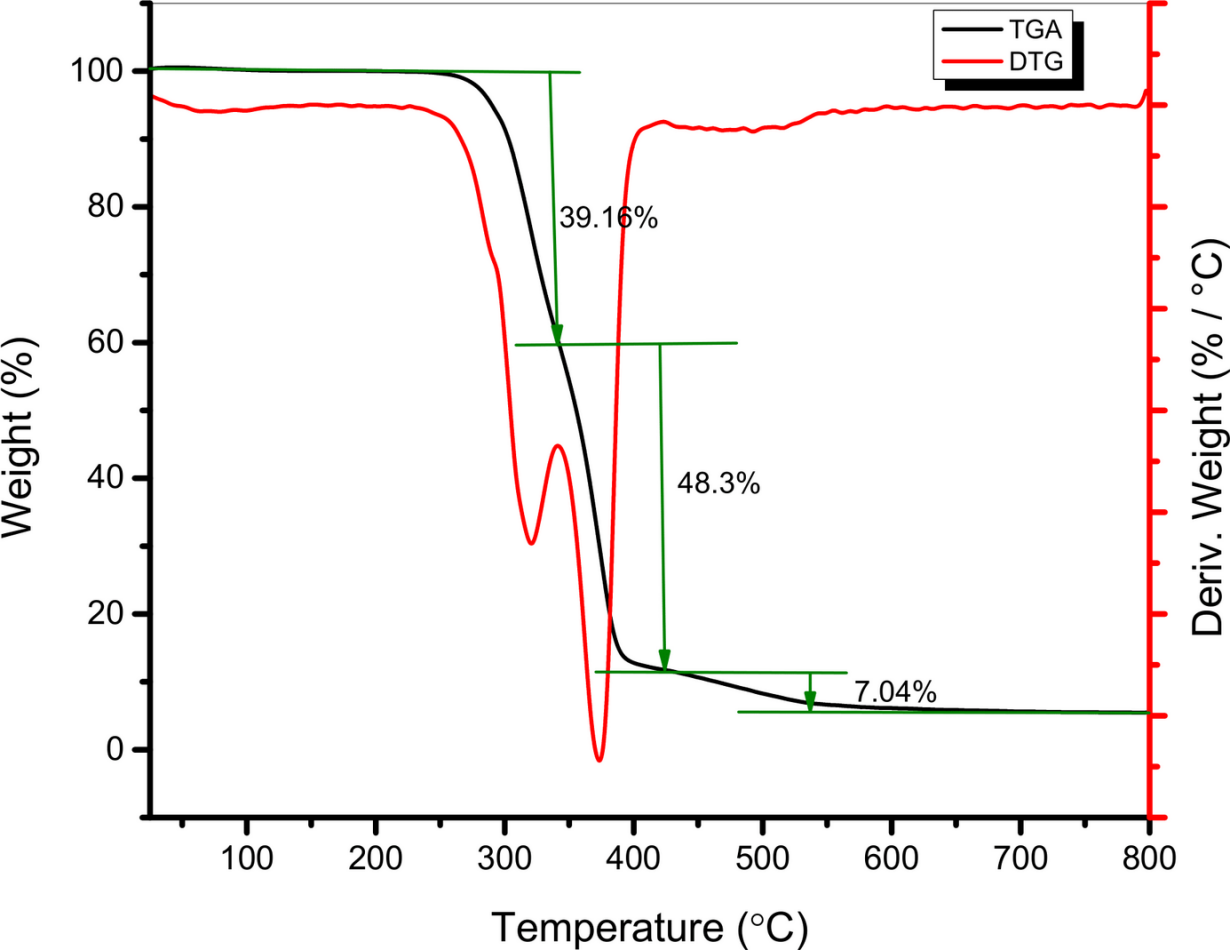
TGA and DTG curves for the title compound.

The thermal properties of the title compound were investigated using thermogravimetric analysis (TGA), derivative thermogravimetry (DTG), and differential scanning calorimetry (DSC). TGA/DTG experiments were carried out under an argon atmosphere by heating crystalline samples from 25 to 800°C at a rate of 10°C min^−1^. The TGA and DTG curves are presented in Fig. 4 ▸.

The TGA profile reveals that the title compound is thermally stable up to 251°C, with no significant mass loss. Above this temperature, three distinct decomposition stages are observed. The first thermal event, occurring between 251 and 343°C, results in a mass loss of 39.16% (calculated: 38.36%) and is attributed to the elimination of a [C_7_H_6_N_2_O_2_] fragment. This transition corresponds to an endothermic peak at 321°C in the DTG curve. The second decomposition step takes place between 343 and 425°C, accompanied by a further mass loss of 48.30% (calculated: 48.59%) and is associated with the release of a [C_14_H_22_] fragment, with a DTG maximum at 372°C. The final thermal degradation occurs between 425 and 800°C, with a final mass loss of 7.04% (calculated: 6.76%), likely due to the liberation of di­nitro­gen (N_2_).

**Figure 5:**
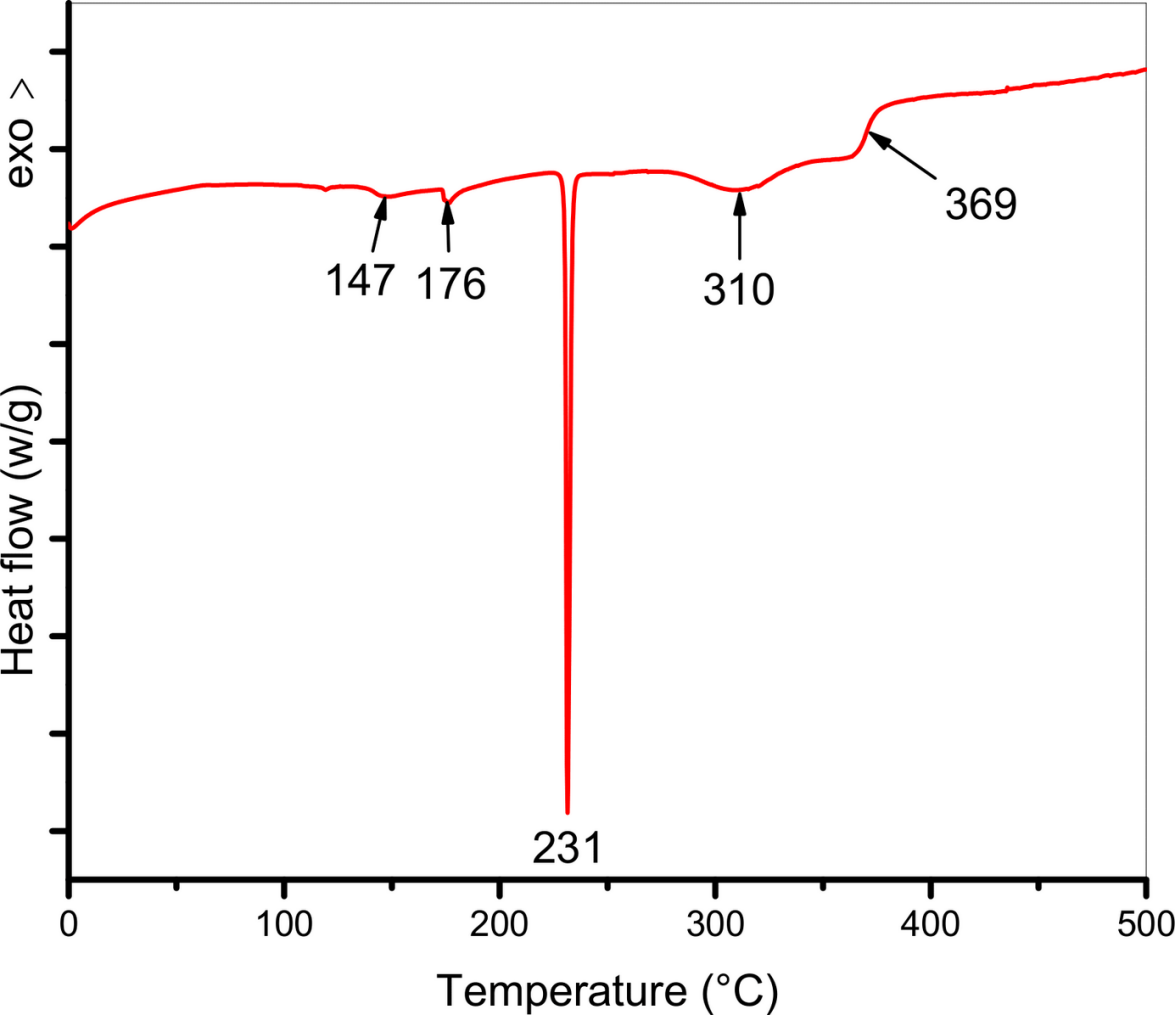
DSC curve of the title compound.

In parallel, DSC measurements were conducted to characterize the thermal transitions of the title compound under similar conditions by heating the sample from 0°C to 500°C at the same rate. The DSC curve in Fig. 5 ▸ reveals four endothermic peaks and one exothermic peak. The first two endothermic signals, located at 147°C (ΔH_1_ = 9.75 Jg^−1^) and 176°C (ΔH_2_ = 7.25 Jg^−1^), exhibit low intensity and are attributed to the release of residual moisture and the desolvation of the sample. A sharp, intense endothermic peak at 231°C (ΔH_f_ = 85.6 Jg^−1^) corresponds to the melting point of the compound, indicating its crystalline nature. Another endothermic transition is observed at 310°C (ΔH_3_ = 56.39 Jg^−1^), marking thermal degradation. Finally, an exothermic event at 369°C (ΔH_4_ = −0.67 Jg^−g^−1^^) is attributed to the advanced decomposition of the acyl hydrazone framework.

## 5. Database survey

A search of the Cambridge Structural Database (version 5.46 with November 2024 updates; Groom *et al.*, 2016 ▸) reveals 88 entries for salicyclic acid acyl­hydrazone derivatives. The most closely related hits are those with a phenyl ring as secondary unit with a hy­droxy group in the *ortho* position and different groups or no groups at all in the *meta* and *para* positions. LUGXUJ (Muthukumar *et al.*, 2020 ▸) has a meth­oxy group in the 5-position with respect to the hy­droxy group in the 2-position; LUGXOD (Muthukumar *et al.*, 2020 ▸) has a meth­oxy group in the *para* position; LUGXET (Muthukumar *et al.*, 2020 ▸) has a meth­oxy group in the 3-position and POJLOR (Mishra *et al.*, 2014 ▸) has no additional groups apart from the hy­droxy group in the *ortho* position. One of the 88 entries (RIYRUN) has an ethanol solvent mol­ecule in its structure, similar to the title compound, but instead of two *tert*-butyl groups in the two *meta* positions and a hy­droxy group in the *ortho* position, it has two meth­oxy groups in the *ortho* and *para* positions of the secondary phenyl unit (Yehye *et al.*, 2008 ▸).

## 6. Synthesis and crystallization

The title compound was synthesized following a reported procedure (Peng *et al.*, 2011 ▸). 2-Hy­droxy­benzohydrazide (0.4 g, 2.6 mmol, 1 equiv.) and 3,5-di-*tert*-butyl-2-hy­droxy­benzaldehyde (0.6 g, 2.6 mmol, 1 equiv.) were dissolved in 10 mL of absolute ethanol, followed by the addition of three drops of glacial acetic acid. The reaction mixture was heated to reflux under continuous stirring for 32 h. After completion, it was allowed to cool to room temperature, then stored in a refrigerator for 2 days to facilitate crystallization. The resulting precipitate was collected by vacuum filtration and thoroughly washed with cold ethanol. The recovered solid was then air-dried. After recrystallization from ethanol, the product was obtained as a white powder. The slow evaporation of the recrystallization filtrate led to the formation of single crystals suitable for X-ray diffraction analysis. Yield 70%; m.p.: 504 K; IR (ATR) cm^−1^: 3221,7 (νO—H), 3078 (νN—H), 1638,3 (νC=O), 1595,8 (νC=N);. ^1^H-NMR (500 MHz, DMSO-*d*_6_), δ (ppm): 12.20 (*s*, 1H), 8.62 (*s*, 1H), 7.87 (*dd*, *J* = 7.9, 1.7 Hz, 1H), 7.45 (*ddd*, *J* = 8.6, 7.2, 1.7 Hz, 1H), 7.32 (*d*, *J* = 2.4 Hz, 1H), 7.24 (*d*, *J* = 2.4 Hz, 1H), 7.02–6.95 (*m*, 2H), 1.41 (*s*, 9H), 1.28 (*s*, 9H); ^13^C-NMR (101 MHz, DMSO-*d*_6_), δ (ppm): 164.51, 159.05, 155.24, 152.47, 140.94, 136.17, 134.45, 129.34, 126.40, 126.25, 119.62, 117.72, 117.41, 116.36, 35.14, 34.37, 31.77, 29.77. UV–vis (DMF): max (nm) = 319, 360.

## 7. Refinement

Crystal data, data collection and structure refinement details are summarized in Table 2 ▸. The solvent mol­ecule was barely visible in the difference-Fourier map, but found to be ethanol, in accordance with the crystallization conditions. It appeared to be disordered over two positions related by an inversion centre and the occupation probability of the ethanol mol­ecule was therefore set at 50%. It was placed using the method described by Kratzert *et al.* (2015 ▸; Kratzert & Krossing, 2018 ▸) using Guzei’s mol­ecular geometry library (Guzei, 2014 ▸) within the *OLEX2* 1.5 inter­face (Dolomanov *et al.*, 2009 ▸) and then refined as a rigid body. The three atoms of the ethanol solvent mol­ecule were refined with equal isotropic displacement parameters. The strongest peaks in the difference-Fourier map are found close to this disordered solvent mol­ecule, proving that its modelling is approximate.

## Supplementary Material

Crystal structure: contains datablock(s) I. DOI: 10.1107/S2056989025006504/ex2092sup1.cif

Structure factors: contains datablock(s) I. DOI: 10.1107/S2056989025006504/ex2092Isup2.hkl

Supporting information file. DOI: 10.1107/S2056989025006504/ex2092Isup3.cml

CCDC reference: 2474602

Additional supporting information:  crystallographic information; 3D view; checkCIF report

## Figures and Tables

**Table 1:** Hydrogen-bond geometry (Å, °)

*D*—H⋯*A*	*D*—H	H⋯*A*	*D*⋯*A*	*D*—H⋯*A*
O46—H46⋯N38	0.84	1.81	2.554 (2)	147
O19—H19⋯N11	0.84	1.87	2.618 (2)	148
O28—H28⋯O36	0.84	1.98	2.696 (2)	142
O28—H28⋯O55	0.84	2.35	2.962 (7)	130
O1—H1⋯O9	0.84	1.85	2.579 (2)	144
N37—H37⋯O9	0.88	2.04	2.905 (2)	168
N10—H10⋯O36^i^	0.88	2.14	2.970 (2)	158

Symmetry code: (i) 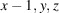.

**Table 2:** Experimental details

Crystal data	
Chemical formula	4C_22_H_28_N_2_O_3_·C_2_H_6_O
*M*_r_	1519.92
Crystal system, space group	Triclinic, *P* 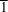
Temperature (K)	173
*a*, *b*, *c* (Å)	9.2163 (4), 15.2640 (7), 15.5406 (7)
α, β, γ (°)	88.046 (2), 76.320 (2), 84.961 (2)
*V* (Å^3^)	2115.79 (17)
*Z*	1
Radiation type	Mo *K*α
μ (mm^−1^)	0.08
Crystal size (mm)	0.41 × 0.12 × 0.09

Data collection	
Diffractometer	Venture Photon-II
Absorption correction	Multi-scan (*SADABS*; Krause *et al.*, 2015 ▸)
*T*_min_, *T*_max_	0.705, 0.745
No. of measured, independent and observed [*I* > 2σ(*I*)] reflections	65507, 8641, 6798
*R*_int_	0.068
(sin θ/λ)_max_ (Å^−1^)	0.625

Refinement	
*R*[*F*^2^ > 2σ(*F*^2^)], *wR*(*F*^2^), *S*	0.060, 0.174, 1.05
No. of reflections	8641
No. of parameters	511
No. of restraints	3
H-atom treatment	H-atom parameters constrained
Δρ_max_, Δρ_min_ (e Å^−3^)	1.56, −0.83

Computer programs: *APEX2* and *SAINT* (Bruker, 2016 ▸), *SUPERFLIP* (Palatinus & Chapuis, 2007 ▸), *SHELXL2018/3* (Sheldrick, 2015 ▸), *Mercury* (Macrae *et al.*, 2020 ▸) and *OLEX2* (Dolomanov *et al.*, 2009 ▸).

## supplementary crystallographic information

### N'-[(E)-3,5-Di-tert-butyl-2-hydroxybenzylidene]-2-hydroxybenzohydrazide ethanol quatersolvate Crystal data

**Table:**

4C_22_H_28_N_2_O_3_·C_2_H_6_O	*F*(000) = 818
*M**_r_* = 1519.92	*D*_x_ = 1.193 Mg m^−^^3^
Triclinic, *P*1	Melting point: 504 K
*a* = 9.2163 (4) Å	Mo *K*α radiation, λ = 0.71073 Å
*b* = 15.2640 (7) Å	Cell parameters from 9976 reflections
*c* = 15.5406 (7) Å	θ = 2.6–26.4°
α = 88.046 (2)°	µ = 0.08 mm^−^^1^
β = 76.320 (2)°	*T* = 173 K
γ = 84.961 (2)°	Prism, clear light colourless
*V* = 2115.79 (17) Å^3^	0.41 × 0.12 × 0.09 mm
*Z* = 1	

### N'-[(E)-3,5-Di-tert-butyl-2-hydroxybenzylidene]-2-hydroxybenzohydrazide ethanol quatersolvate Data collection

**Table:**

Venture Photon-II diffractometer	6798 reflections with *I* > 2σ(*I*)
φ and ω scans	*R*_int_ = 0.068
Absorption correction: multi-scan (SADABS; Krause *et al.*, 2015)	θ_max_ = 26.4°, θ_min_ = 1.9°
*T*_min_ = 0.705, *T*_max_ = 0.745	*h* = −11→11
65507 measured reflections	*k* = −19→19
8641 independent reflections	*l* = −19→19

### N'-[(E)-3,5-Di-tert-butyl-2-hydroxybenzylidene]-2-hydroxybenzohydrazide ethanol quatersolvate Refinement

**Table:**

Refinement on *F*^2^	Primary atom site location: dual
Least-squares matrix: full	Hydrogen site location: mixed
*R*[*F*^2^ > 2σ(*F*^2^)] = 0.060	H-atom parameters constrained
*wR*(*F*^2^) = 0.174	*w* = 1/[σ^2^(*F*_o_^2^) + (0.0809*P*)^2^ + 1.8079*P*] where *P* = (*F*_o_^2^ + 2*F*_c_^2^)/3
*S* = 1.05	(Δ/σ)_max_ < 0.001
8641 reflections	Δρ_max_ = 1.56 e Å^−^^3^
511 parameters	Δρ_min_ = −0.83 e Å^−^^3^
3 restraints	

### N'-[(E)-3,5-Di-tert-butyl-2-hydroxybenzylidene]-2-hydroxybenzohydrazide ethanol quatersolvate Special details

**Table:**

Geometry. All esds (except the esd in the dihedral angle between two l.s. planes) are estimated using the full covariance matrix. The cell esds are taken into account individually in the estimation of esds in distances, angles and torsion angles; correlations between esds in cell parameters are only used when they are defined by crystal symmetry. An approximate (isotropic) treatment of cell esds is used for estimating esds involving l.s. planes.

### N'-[(E)-3,5-Di-tert-butyl-2-hydroxybenzylidene]-2-hydroxybenzohydrazide ethanol quatersolvate Fractional atomic coordinates and isotropic or equivalent isotropic displacement parameters (Å^2^)

**Table:**

	*x*	*y*	*z*	*U*_iso_*/*U*_eq_	Occ. (<1)
O46	0.65865 (16)	0.26684 (10)	0.73467 (9)	0.0258 (3)	
H46	0.635044	0.260176	0.686443	0.039*	
O36	0.72292 (16)	0.25601 (10)	0.48969 (9)	0.0280 (3)	
O9	0.22792 (16)	0.12038 (10)	0.53659 (10)	0.0305 (3)	
O19	0.17769 (17)	0.32035 (9)	0.70208 (11)	0.0307 (3)	
H19	0.164767	0.288120	0.662233	0.046*	
N38	0.51741 (18)	0.20827 (11)	0.62873 (10)	0.0230 (4)	
O28	0.75909 (19)	0.30941 (11)	0.31935 (10)	0.0372 (4)	
H28	0.764336	0.315344	0.372038	0.056*	
O1	0.28536 (18)	−0.03034 (10)	0.46044 (12)	0.0379 (4)	
H1	0.283051	0.005394	0.500761	0.057*	
N37	0.50209 (18)	0.19703 (11)	0.54418 (10)	0.0225 (3)	
H37	0.420661	0.177496	0.534135	0.027*	
N11	0.04910 (19)	0.26608 (11)	0.58262 (11)	0.0252 (4)	
N10	0.02547 (19)	0.20880 (11)	0.52166 (11)	0.0249 (4)	
H10	−0.051396	0.219062	0.497218	0.030*	
C42	0.5841 (2)	0.22838 (12)	0.88740 (12)	0.0210 (4)	
C40	0.4465 (2)	0.18053 (13)	0.78331 (13)	0.0220 (4)	
C41	0.5635 (2)	0.22580 (12)	0.80070 (12)	0.0207 (4)	
C50	0.7201 (2)	0.26530 (15)	1.00432 (13)	0.0283 (4)	
H50A	0.627382	0.292933	1.041359	0.042*	
H50B	0.805848	0.294201	1.013730	0.042*	
H50C	0.730817	0.202806	1.020324	0.042*	
C35	0.6169 (2)	0.21716 (12)	0.47732 (13)	0.0219 (4)	
C29	0.6872 (2)	0.23731 (14)	0.31338 (13)	0.0257 (4)	
C39	0.4263 (2)	0.17367 (13)	0.69378 (13)	0.0232 (4)	
H39	0.346013	0.143697	0.683549	0.028*	
C44	0.3639 (2)	0.14244 (13)	0.93961 (13)	0.0230 (4)	
C34	0.6116 (2)	0.19024 (13)	0.38736 (12)	0.0224 (4)	
C45	0.3489 (2)	0.13987 (13)	0.85324 (13)	0.0236 (4)	
H45	0.270213	0.109758	0.840996	0.028*	
C47	0.7144 (2)	0.27460 (13)	0.90683 (12)	0.0225 (4)	
C13	−0.0273 (2)	0.40263 (13)	0.65791 (13)	0.0241 (4)	
C31	0.6144 (3)	0.13871 (17)	0.21695 (14)	0.0366 (5)	
H31	0.614724	0.121507	0.158726	0.044*	
C8	0.1212 (2)	0.13701 (13)	0.49994 (13)	0.0231 (4)	
C43	0.4825 (2)	0.18704 (13)	0.95389 (12)	0.0226 (4)	
H43	0.494428	0.189275	1.012819	0.027*	
C30	0.6871 (3)	0.21060 (16)	0.22861 (14)	0.0329 (5)	
H30	0.737790	0.242345	0.178444	0.039*	
C12	−0.0410 (2)	0.33633 (13)	0.59621 (14)	0.0257 (4)	
H12	−0.118364	0.344935	0.565058	0.031*	
C7	0.0964 (2)	0.07976 (13)	0.43073 (13)	0.0239 (4)	
C14	0.0804 (2)	0.39356 (13)	0.70912 (14)	0.0238 (4)	
C18	−0.1271 (2)	0.47858 (13)	0.66560 (14)	0.0269 (4)	
H18	−0.199579	0.484258	0.630863	0.032*	
C6	−0.0087 (2)	0.10249 (13)	0.38008 (14)	0.0266 (4)	
H6	−0.066982	0.157270	0.389977	0.032*	
C17	−0.1221 (2)	0.54490 (13)	0.72237 (14)	0.0270 (4)	
C51	0.2515 (2)	0.10209 (14)	1.01679 (14)	0.0278 (4)	
C24	−0.2303 (2)	0.62820 (14)	0.73318 (16)	0.0334 (5)	
C15	0.0893 (2)	0.46009 (13)	0.76769 (14)	0.0254 (4)	
C49	0.8644 (2)	0.23250 (14)	0.85241 (14)	0.0278 (4)	
H49A	0.874579	0.169835	0.867832	0.042*	
H49B	0.947070	0.261634	0.865589	0.042*	
H49C	0.866967	0.239203	0.789133	0.042*	
C5	−0.0298 (3)	0.04778 (15)	0.31646 (15)	0.0336 (5)	
H5	−0.102623	0.064206	0.283403	0.040*	
C20	0.2080 (3)	0.45295 (14)	0.82303 (15)	0.0311 (5)	
C16	−0.0131 (2)	0.53339 (13)	0.77192 (14)	0.0270 (4)	
H16	−0.008723	0.578850	0.811259	0.032*	
C33	0.5407 (2)	0.11667 (14)	0.37355 (14)	0.0293 (4)	
H33	0.491580	0.083630	0.423191	0.035*	
C2	0.1826 (2)	−0.00160 (13)	0.41399 (14)	0.0273 (4)	
C32	0.5402 (3)	0.09079 (16)	0.28930 (15)	0.0357 (5)	
H32	0.490113	0.041092	0.280915	0.043*	
C4	0.0570 (3)	−0.03212 (15)	0.30105 (16)	0.0365 (5)	
H4	0.043646	−0.070171	0.256931	0.044*	
C48	0.6972 (3)	0.37348 (14)	0.88509 (15)	0.0308 (5)	
H48A	0.697215	0.381704	0.822269	0.046*	
H48B	0.780871	0.402081	0.897729	0.046*	
H48C	0.602563	0.399657	0.921413	0.046*	
C3	0.1618 (3)	−0.05629 (14)	0.34911 (16)	0.0351 (5)	
H3	0.220444	−0.110848	0.337886	0.042*	
C23	0.3653 (3)	0.44539 (18)	0.76081 (18)	0.0426 (6)	
H23A	0.377242	0.392668	0.724717	0.064*	
H23B	0.440997	0.441155	0.796017	0.064*	
H23C	0.377915	0.497535	0.722040	0.064*	
C21	0.1973 (3)	0.53449 (16)	0.88016 (18)	0.0446 (6)	
H21A	0.210471	0.586969	0.841893	0.067*	
H21B	0.275799	0.527964	0.913388	0.067*	
H21C	0.098838	0.540563	0.921634	0.067*	
C22	0.1879 (3)	0.37306 (15)	0.88618 (16)	0.0372 (5)	
H22A	0.089266	0.379968	0.927368	0.056*	
H22B	0.266156	0.368743	0.919611	0.056*	
H22C	0.195591	0.319464	0.851863	0.056*	
C25	−0.3314 (3)	0.62972 (19)	0.8263 (2)	0.0585 (8)	
H25A	−0.391140	0.578717	0.835110	0.088*	
H25B	−0.398336	0.683877	0.833846	0.088*	
H25C	−0.269707	0.627553	0.869914	0.088*	
C52	0.3118 (3)	0.0883 (2)	1.10016 (17)	0.0523 (8)	
H52A	0.406888	0.051682	1.086298	0.079*	
H52B	0.239179	0.059091	1.146016	0.079*	
H52C	0.328047	0.145460	1.121664	0.079*	
C26	−0.1421 (3)	0.70996 (16)	0.7183 (2)	0.0489 (7)	
H26A	−0.083670	0.711464	0.763300	0.073*	
H26B	−0.211848	0.762805	0.722806	0.073*	
H26C	−0.074210	0.707985	0.659299	0.073*	
C27	−0.3284 (3)	0.63290 (19)	0.6662 (2)	0.0608 (8)	
H27A	−0.264485	0.629885	0.606056	0.091*	
H27B	−0.391877	0.688367	0.673078	0.091*	
H27C	−0.391661	0.583452	0.676515	0.091*	
C54	0.1086 (3)	0.1626 (2)	1.0375 (2)	0.0561 (8)	
H54A	0.130545	0.220013	1.055353	0.084*	
H54B	0.035254	0.136947	1.085817	0.084*	
H54C	0.067656	0.169923	0.984729	0.084*	
C53	0.2168 (4)	0.01109 (19)	0.99214 (19)	0.0549 (8)	
H53A	0.173153	0.016627	0.940293	0.082*	
H53B	0.145452	−0.013806	1.042042	0.082*	
H53C	0.309490	−0.027725	0.978326	0.082*	
O55	0.7784 (6)	0.4436 (5)	0.4486 (5)	0.1154 (16)*	0.5
H55	0.763376	0.392417	0.489724	0.173*	0.5
C56	0.6508 (8)	0.5057 (4)	0.4701 (7)	0.1154 (16)*	0.5
H56A	0.637535	0.539211	0.416475	0.138*	0.5
H56B	0.663717	0.547606	0.514391	0.138*	0.5
C57	0.5162 (6)	0.4546 (6)	0.5074 (8)	0.1154 (16)*	0.5
H57A	0.504651	0.413327	0.462986	0.173*	0.5
H57B	0.426136	0.495496	0.522735	0.173*	0.5
H57C	0.530406	0.421957	0.560532	0.173*	0.5

### N'-[(E)-3,5-Di-tert-butyl-2-hydroxybenzylidene]-2-hydroxybenzohydrazide ethanol quatersolvate Atomic displacement parameters (Å^2^)

**Table:**

	*U*^11^	*U*^22^	*U*^33^	*U*^12^	*U*^13^	*U*^23^
O46	0.0277 (7)	0.0340 (8)	0.0164 (7)	−0.0086 (6)	−0.0049 (6)	0.0023 (6)
O36	0.0264 (7)	0.0335 (8)	0.0255 (7)	−0.0045 (6)	−0.0076 (6)	−0.0057 (6)
O9	0.0235 (7)	0.0315 (8)	0.0375 (9)	−0.0002 (6)	−0.0101 (6)	−0.0004 (6)
O19	0.0305 (8)	0.0245 (7)	0.0396 (9)	0.0077 (6)	−0.0155 (7)	−0.0095 (6)
N38	0.0240 (8)	0.0286 (9)	0.0170 (8)	0.0033 (7)	−0.0075 (6)	−0.0036 (6)
O28	0.0457 (10)	0.0398 (9)	0.0280 (8)	−0.0152 (7)	−0.0089 (7)	0.0051 (7)
O1	0.0355 (9)	0.0280 (8)	0.0480 (10)	0.0088 (7)	−0.0095 (7)	0.0000 (7)
N37	0.0212 (8)	0.0311 (9)	0.0167 (8)	0.0004 (7)	−0.0075 (6)	−0.0030 (6)
N11	0.0267 (9)	0.0237 (8)	0.0260 (9)	−0.0020 (7)	−0.0070 (7)	−0.0043 (7)
N10	0.0244 (8)	0.0247 (8)	0.0266 (9)	0.0008 (7)	−0.0085 (7)	−0.0052 (7)
C42	0.0228 (9)	0.0215 (9)	0.0188 (9)	−0.0017 (7)	−0.0049 (7)	−0.0008 (7)
C40	0.0214 (9)	0.0246 (9)	0.0197 (9)	0.0009 (7)	−0.0050 (7)	−0.0020 (7)
C41	0.0212 (9)	0.0220 (9)	0.0175 (9)	−0.0008 (7)	−0.0025 (7)	0.0004 (7)
C50	0.0319 (11)	0.0344 (11)	0.0206 (10)	−0.0086 (9)	−0.0080 (8)	−0.0020 (8)
C35	0.0221 (9)	0.0229 (9)	0.0207 (9)	0.0031 (7)	−0.0067 (7)	−0.0015 (7)
C29	0.0243 (10)	0.0292 (10)	0.0236 (10)	0.0011 (8)	−0.0075 (8)	0.0021 (8)
C39	0.0222 (9)	0.0265 (10)	0.0219 (10)	0.0008 (7)	−0.0077 (8)	−0.0030 (8)
C44	0.0221 (9)	0.0237 (9)	0.0219 (10)	−0.0020 (7)	−0.0030 (8)	0.0013 (7)
C34	0.0209 (9)	0.0282 (10)	0.0178 (9)	0.0020 (7)	−0.0055 (7)	−0.0016 (7)
C45	0.0211 (9)	0.0258 (10)	0.0246 (10)	−0.0035 (7)	−0.0060 (8)	−0.0017 (8)
C47	0.0262 (10)	0.0243 (10)	0.0180 (9)	−0.0062 (8)	−0.0058 (7)	−0.0002 (7)
C13	0.0241 (10)	0.0207 (9)	0.0273 (10)	−0.0009 (7)	−0.0058 (8)	−0.0017 (8)
C31	0.0405 (13)	0.0503 (14)	0.0200 (10)	0.0016 (11)	−0.0101 (9)	−0.0071 (9)
C8	0.0206 (9)	0.0227 (9)	0.0245 (10)	−0.0036 (7)	−0.0023 (7)	0.0029 (8)
C43	0.0269 (10)	0.0253 (10)	0.0158 (9)	−0.0028 (8)	−0.0049 (7)	−0.0001 (7)
C30	0.0359 (12)	0.0439 (13)	0.0182 (10)	−0.0009 (10)	−0.0066 (9)	0.0032 (9)
C12	0.0239 (10)	0.0269 (10)	0.0267 (10)	−0.0002 (8)	−0.0074 (8)	−0.0008 (8)
C7	0.0230 (10)	0.0197 (9)	0.0255 (10)	−0.0025 (7)	0.0015 (8)	0.0002 (7)
C14	0.0217 (9)	0.0198 (9)	0.0291 (10)	0.0009 (7)	−0.0053 (8)	−0.0009 (8)
C18	0.0232 (10)	0.0250 (10)	0.0321 (11)	0.0011 (8)	−0.0072 (8)	0.0011 (8)
C6	0.0269 (10)	0.0236 (10)	0.0275 (10)	−0.0002 (8)	−0.0028 (8)	−0.0035 (8)
C17	0.0233 (10)	0.0205 (9)	0.0339 (11)	−0.0002 (8)	−0.0006 (8)	0.0007 (8)
C51	0.0239 (10)	0.0340 (11)	0.0242 (10)	−0.0074 (8)	−0.0020 (8)	0.0049 (8)
C24	0.0274 (11)	0.0224 (10)	0.0462 (13)	0.0037 (8)	−0.0026 (9)	−0.0002 (9)
C15	0.0255 (10)	0.0224 (10)	0.0284 (10)	−0.0026 (8)	−0.0063 (8)	−0.0014 (8)
C49	0.0266 (10)	0.0340 (11)	0.0237 (10)	−0.0052 (8)	−0.0068 (8)	−0.0009 (8)
C5	0.0359 (12)	0.0337 (12)	0.0308 (11)	−0.0012 (9)	−0.0067 (9)	−0.0066 (9)
C20	0.0340 (11)	0.0275 (11)	0.0358 (12)	−0.0032 (9)	−0.0153 (9)	−0.0042 (9)
C16	0.0280 (10)	0.0202 (9)	0.0314 (11)	−0.0027 (8)	−0.0037 (8)	−0.0033 (8)
C33	0.0318 (11)	0.0327 (11)	0.0231 (10)	−0.0046 (9)	−0.0050 (8)	−0.0037 (8)
C2	0.0240 (10)	0.0206 (9)	0.0327 (11)	−0.0009 (8)	0.0019 (8)	0.0024 (8)
C32	0.0391 (13)	0.0412 (13)	0.0286 (11)	−0.0082 (10)	−0.0085 (9)	−0.0093 (9)
C4	0.0444 (13)	0.0288 (11)	0.0327 (12)	−0.0057 (10)	0.0004 (10)	−0.0098 (9)
C48	0.0384 (12)	0.0255 (10)	0.0304 (11)	−0.0079 (9)	−0.0104 (9)	0.0009 (8)
C3	0.0363 (12)	0.0219 (10)	0.0398 (13)	0.0012 (9)	0.0050 (10)	−0.0041 (9)
C23	0.0303 (12)	0.0474 (14)	0.0542 (16)	−0.0072 (10)	−0.0163 (11)	−0.0012 (12)
C21	0.0599 (16)	0.0321 (12)	0.0512 (15)	−0.0049 (11)	−0.0298 (13)	−0.0092 (11)
C22	0.0454 (14)	0.0327 (12)	0.0385 (13)	−0.0027 (10)	−0.0199 (11)	−0.0007 (10)
C25	0.0465 (16)	0.0405 (15)	0.071 (2)	0.0085 (12)	0.0173 (14)	−0.0001 (14)
C52	0.0432 (15)	0.083 (2)	0.0324 (13)	−0.0252 (14)	−0.0084 (11)	0.0258 (13)
C26	0.0444 (15)	0.0256 (12)	0.0713 (19)	0.0013 (10)	−0.0047 (13)	0.0030 (12)
C27	0.0497 (17)	0.0428 (15)	0.094 (2)	0.0192 (13)	−0.0315 (16)	−0.0065 (15)
C54	0.0370 (14)	0.0650 (19)	0.0510 (17)	0.0080 (13)	0.0127 (12)	0.0196 (14)
C53	0.0684 (19)	0.0484 (16)	0.0440 (15)	−0.0295 (14)	0.0032 (13)	0.0036 (12)

### N'-[(E)-3,5-Di-tert-butyl-2-hydroxybenzylidene]-2-hydroxybenzohydrazide ethanol quatersolvate Geometric parameters (Å, º)

**Table:**

O46—H46	0.8400	C24—C25	1.525 (4)
O46—C41	1.357 (2)	C24—C26	1.530 (3)
O36—C35	1.242 (2)	C24—C27	1.528 (4)
O9—C8	1.252 (2)	C15—C20	1.538 (3)
O19—H19	0.8400	C15—C16	1.392 (3)
O19—C14	1.360 (2)	C49—H49A	0.9800
N38—N37	1.372 (2)	C49—H49B	0.9800
N38—C39	1.283 (3)	C49—H49C	0.9800
O28—H28	0.8400	C5—H5	0.9500
O28—C29	1.350 (3)	C5—C4	1.394 (3)
O1—H1	0.8400	C20—C23	1.538 (3)
O1—C2	1.356 (3)	C20—C21	1.536 (3)
N37—H37	0.8800	C20—C22	1.535 (3)
N37—C35	1.345 (3)	C16—H16	0.9500
N11—N10	1.377 (2)	C33—H33	0.9500
N11—C12	1.288 (3)	C33—C32	1.382 (3)
N10—H10	0.8800	C2—C3	1.388 (3)
N10—C8	1.344 (3)	C32—H32	0.9500
C42—C41	1.407 (3)	C4—H4	0.9500
C42—C47	1.538 (3)	C4—C3	1.373 (4)
C42—C43	1.395 (3)	C48—H48A	0.9800
C40—C41	1.412 (3)	C48—H48B	0.9800
C40—C39	1.455 (3)	C48—H48C	0.9800
C40—C45	1.402 (3)	C3—H3	0.9500
C50—H50A	0.9800	C23—H23A	0.9800
C50—H50B	0.9800	C23—H23B	0.9800
C50—H50C	0.9800	C23—H23C	0.9800
C50—C47	1.530 (3)	C21—H21A	0.9800
C35—C34	1.483 (3)	C21—H21B	0.9800
C29—C34	1.406 (3)	C21—H21C	0.9800
C29—C30	1.392 (3)	C22—H22A	0.9800
C39—H39	0.9500	C22—H22B	0.9800
C44—C45	1.384 (3)	C22—H22C	0.9800
C44—C43	1.401 (3)	C25—H25A	0.9800
C44—C51	1.538 (3)	C25—H25B	0.9800
C34—C33	1.395 (3)	C25—H25C	0.9800
C45—H45	0.9500	C52—H52A	0.9800
C47—C49	1.538 (3)	C52—H52B	0.9800
C47—C48	1.538 (3)	C52—H52C	0.9800
C13—C12	1.451 (3)	C26—H26A	0.9800
C13—C14	1.407 (3)	C26—H26B	0.9800
C13—C18	1.405 (3)	C26—H26C	0.9800
C31—H31	0.9500	C27—H27A	0.9800
C31—C30	1.373 (3)	C27—H27B	0.9800
C31—C32	1.391 (3)	C27—H27C	0.9800
C8—C7	1.479 (3)	C54—H54A	0.9800
C43—H43	0.9500	C54—H54B	0.9800
C30—H30	0.9500	C54—H54C	0.9800
C12—H12	0.9500	C53—H53A	0.9800
C7—C6	1.400 (3)	C53—H53B	0.9800
C7—C2	1.411 (3)	C53—H53C	0.9800
C14—C15	1.407 (3)	O55—H55	0.9909
C18—H18	0.9500	O55—C56	1.4251
C18—C17	1.376 (3)	C56—H56A	0.9900
C6—H6	0.9500	C56—H56B	0.9900
C6—C5	1.374 (3)	C56—C57	1.5101
C17—C24	1.533 (3)	C56—H57B^i^	0.693 (14)
C17—C16	1.400 (3)	C57—H57A	0.9800
C51—C52	1.528 (3)	C57—H57B^i^	1.005 (16)
C51—C54	1.515 (3)	C57—H57B	0.9800
C51—C53	1.533 (3)	C57—H57C	0.9800
			
C41—O46—H46	109.5	C4—C5—H5	120.4
C14—O19—H19	109.5	C23—C20—C15	109.43 (19)
C39—N38—N37	119.06 (17)	C21—C20—C15	111.95 (18)
C29—O28—H28	109.5	C21—C20—C23	107.3 (2)
C2—O1—H1	109.5	C22—C20—C15	110.73 (18)
N38—N37—H37	121.4	C22—C20—C23	110.2 (2)
C35—N37—N38	117.12 (16)	C22—C20—C21	107.1 (2)
C35—N37—H37	121.4	C17—C16—H16	117.7
C12—N11—N10	116.05 (17)	C15—C16—C17	124.67 (19)
N11—N10—H10	120.4	C15—C16—H16	117.7
C8—N10—N11	119.23 (17)	C34—C33—H33	119.3
C8—N10—H10	120.4	C32—C33—C34	121.5 (2)
C41—C42—C47	120.77 (16)	C32—C33—H33	119.3
C43—C42—C41	117.20 (17)	O1—C2—C7	122.4 (2)
C43—C42—C47	122.03 (17)	O1—C2—C3	117.49 (19)
C41—C40—C39	121.40 (17)	C3—C2—C7	120.1 (2)
C45—C40—C41	119.66 (17)	C31—C32—H32	120.6
C45—C40—C39	118.93 (18)	C33—C32—C31	118.9 (2)
O46—C41—C42	118.75 (17)	C33—C32—H32	120.6
O46—C41—C40	121.08 (17)	C5—C4—H4	119.7
C42—C41—C40	120.17 (17)	C3—C4—C5	120.5 (2)
H50A—C50—H50B	109.5	C3—C4—H4	119.7
H50A—C50—H50C	109.5	C47—C48—H48A	109.5
H50B—C50—H50C	109.5	C47—C48—H48B	109.5
C47—C50—H50A	109.5	C47—C48—H48C	109.5
C47—C50—H50B	109.5	H48A—C48—H48B	109.5
C47—C50—H50C	109.5	H48A—C48—H48C	109.5
O36—C35—N37	122.08 (18)	H48B—C48—H48C	109.5
O36—C35—C34	121.20 (17)	C2—C3—H3	119.8
N37—C35—C34	116.72 (17)	C4—C3—C2	120.5 (2)
O28—C29—C34	123.55 (18)	C4—C3—H3	119.8
O28—C29—C30	116.96 (19)	C20—C23—H23A	109.5
C30—C29—C34	119.5 (2)	C20—C23—H23B	109.5
N38—C39—C40	119.71 (18)	C20—C23—H23C	109.5
N38—C39—H39	120.1	H23A—C23—H23B	109.5
C40—C39—H39	120.1	H23A—C23—H23C	109.5
C45—C44—C43	116.79 (17)	H23B—C23—H23C	109.5
C45—C44—C51	121.59 (18)	C20—C21—H21A	109.5
C43—C44—C51	121.56 (18)	C20—C21—H21B	109.5
C29—C34—C35	119.12 (18)	C20—C21—H21C	109.5
C33—C34—C35	122.01 (18)	H21A—C21—H21B	109.5
C33—C34—C29	118.78 (18)	H21A—C21—H21C	109.5
C40—C45—H45	119.1	H21B—C21—H21C	109.5
C44—C45—C40	121.77 (18)	C20—C22—H22A	109.5
C44—C45—H45	119.1	C20—C22—H22B	109.5
C50—C47—C42	111.90 (16)	C20—C22—H22C	109.5
C50—C47—C49	106.85 (16)	H22A—C22—H22B	109.5
C50—C47—C48	107.49 (16)	H22A—C22—H22C	109.5
C49—C47—C42	109.78 (16)	H22B—C22—H22C	109.5
C48—C47—C42	110.32 (16)	C24—C25—H25A	109.5
C48—C47—C49	110.43 (17)	C24—C25—H25B	109.5
C14—C13—C12	122.84 (18)	C24—C25—H25C	109.5
C18—C13—C12	117.43 (18)	H25A—C25—H25B	109.5
C18—C13—C14	119.73 (18)	H25A—C25—H25C	109.5
C30—C31—H31	119.6	H25B—C25—H25C	109.5
C30—C31—C32	120.8 (2)	C51—C52—H52A	109.5
C32—C31—H31	119.6	C51—C52—H52B	109.5
O9—C8—N10	120.77 (19)	C51—C52—H52C	109.5
O9—C8—C7	121.74 (18)	H52A—C52—H52B	109.5
N10—C8—C7	117.48 (17)	H52A—C52—H52C	109.5
C42—C43—C44	124.38 (18)	H52B—C52—H52C	109.5
C42—C43—H43	117.8	C24—C26—H26A	109.5
C44—C43—H43	117.8	C24—C26—H26B	109.5
C29—C30—H30	119.7	C24—C26—H26C	109.5
C31—C30—C29	120.5 (2)	H26A—C26—H26B	109.5
C31—C30—H30	119.7	H26A—C26—H26C	109.5
N11—C12—C13	121.64 (19)	H26B—C26—H26C	109.5
N11—C12—H12	119.2	C24—C27—H27A	109.5
C13—C12—H12	119.2	C24—C27—H27B	109.5
C6—C7—C8	123.11 (18)	C24—C27—H27C	109.5
C6—C7—C2	117.86 (19)	H27A—C27—H27B	109.5
C2—C7—C8	119.04 (19)	H27A—C27—H27C	109.5
O19—C14—C13	120.19 (18)	H27B—C27—H27C	109.5
O19—C14—C15	119.30 (18)	C51—C54—H54A	109.5
C13—C14—C15	120.51 (18)	C51—C54—H54B	109.5
C13—C18—H18	119.3	C51—C54—H54C	109.5
C17—C18—C13	121.42 (19)	H54A—C54—H54B	109.5
C17—C18—H18	119.3	H54A—C54—H54C	109.5
C7—C6—H6	119.1	H54B—C54—H54C	109.5
C5—C6—C7	121.8 (2)	C51—C53—H53A	109.5
C5—C6—H6	119.1	C51—C53—H53B	109.5
C18—C17—C24	122.8 (2)	C51—C53—H53C	109.5
C18—C17—C16	117.03 (18)	H53A—C53—H53B	109.5
C16—C17—C24	120.13 (19)	H53A—C53—H53C	109.5
C52—C51—C44	112.53 (17)	H53B—C53—H53C	109.5
C52—C51—C53	106.2 (2)	C56—O55—H55	109.5
C54—C51—C44	108.71 (18)	O55—C56—H56A	110.2
C54—C51—C52	109.2 (2)	O55—C56—H56B	110.2
C54—C51—C53	109.7 (2)	O55—C56—C57	107.4
C53—C51—C44	110.49 (18)	O55—C56—H57B^i^	136 (5)
C25—C24—C17	109.49 (19)	H56A—C56—H56B	108.5
C25—C24—C26	109.3 (2)	H56A—C56—H57B^i^	82.3
C25—C24—C27	108.7 (2)	H56B—C56—H57B^i^	104.3
C26—C24—C17	110.01 (18)	C57—C56—H56A	110.2
C27—C24—C17	111.9 (2)	C57—C56—H56B	110.2
C27—C24—C26	107.3 (2)	C57—C56—H57B^i^	33.2 (13)
C14—C15—C20	121.70 (18)	C56—C57—H57A	109.5
C16—C15—C14	116.63 (19)	C56—C57—H57B^i^	22.2 (7)
C16—C15—C20	121.67 (18)	C56—C57—H57B	109.5
C47—C49—H49A	109.5	C56—C57—H57C	109.5
C47—C49—H49B	109.5	H57A—C57—H57B^i^	109.8
C47—C49—H49C	109.5	H57A—C57—H57B	109.5
H49A—C49—H49B	109.5	H57A—C57—H57C	109.5
H49A—C49—H49C	109.5	H57B—C57—H57B^i^	89.2
H49B—C49—H49C	109.5	H57B—C57—H57C	109.5
C6—C5—H5	120.4	H57C—C57—H57B^i^	127.0
C6—C5—C4	119.2 (2)		
			
O36—C35—C34—C29	−26.1 (3)	C8—C7—C2—O1	1.5 (3)
O36—C35—C34—C33	150.3 (2)	C8—C7—C2—C3	−179.73 (18)
O9—C8—C7—C6	−171.03 (19)	C43—C42—C41—O46	178.74 (17)
O9—C8—C7—C2	8.6 (3)	C43—C42—C41—C40	−1.8 (3)
O19—C14—C15—C20	1.0 (3)	C43—C42—C47—C50	2.5 (3)
O19—C14—C15—C16	179.95 (18)	C43—C42—C47—C49	121.0 (2)
N38—N37—C35—O36	−10.2 (3)	C43—C42—C47—C48	−117.1 (2)
N38—N37—C35—C34	169.49 (16)	C43—C44—C45—C40	−0.5 (3)
O28—C29—C34—C35	−3.6 (3)	C43—C44—C51—C52	−19.5 (3)
O28—C29—C34—C33	179.85 (19)	C43—C44—C51—C54	101.6 (2)
O28—C29—C30—C31	−178.9 (2)	C43—C44—C51—C53	−138.0 (2)
O1—C2—C3—C4	178.5 (2)	C30—C29—C34—C35	177.79 (18)
N37—N38—C39—C40	176.36 (16)	C30—C29—C34—C33	1.2 (3)
N37—C35—C34—C29	154.17 (18)	C30—C31—C32—C33	0.0 (4)
N37—C35—C34—C33	−29.4 (3)	C12—N11—N10—C8	175.70 (18)
N11—N10—C8—O9	2.4 (3)	C12—C13—C14—O19	0.1 (3)
N11—N10—C8—C7	−176.81 (16)	C12—C13—C14—C15	179.71 (19)
N10—N11—C12—C13	−179.22 (17)	C12—C13—C18—C17	−179.93 (19)
N10—C8—C7—C6	8.2 (3)	C7—C6—C5—C4	−0.8 (3)
N10—C8—C7—C2	−172.16 (17)	C7—C2—C3—C4	−0.3 (3)
C41—C42—C47—C50	−177.05 (17)	C14—C13—C12—N11	−2.4 (3)
C41—C42—C47—C49	−58.6 (2)	C14—C13—C18—C17	0.1 (3)
C41—C42—C47—C48	63.3 (2)	C14—C15—C20—C23	59.9 (3)
C41—C42—C43—C44	1.0 (3)	C14—C15—C20—C21	178.7 (2)
C41—C40—C39—N38	1.0 (3)	C14—C15—C20—C22	−61.8 (3)
C41—C40—C45—C44	−0.2 (3)	C14—C15—C16—C17	−0.1 (3)
C35—C34—C33—C32	−178.1 (2)	C18—C13—C12—N11	177.55 (19)
C29—C34—C33—C32	−1.6 (3)	C18—C13—C14—O19	−179.92 (18)
C39—N38—N37—C35	−165.93 (17)	C18—C13—C14—C15	−0.3 (3)
C39—C40—C41—O46	2.0 (3)	C18—C17—C24—C25	113.7 (3)
C39—C40—C41—C42	−177.45 (17)	C18—C17—C24—C26	−126.1 (2)
C39—C40—C45—C44	178.68 (18)	C18—C17—C24—C27	−7.0 (3)
C34—C29—C30—C31	−0.2 (3)	C18—C17—C16—C15	−0.1 (3)
C34—C33—C32—C31	1.0 (3)	C6—C7—C2—O1	−178.82 (18)
C45—C40—C41—O46	−179.09 (17)	C6—C7—C2—C3	−0.1 (3)
C45—C40—C41—C42	1.4 (3)	C6—C5—C4—C3	0.4 (3)
C45—C40—C39—N38	−177.95 (18)	C51—C44—C45—C40	176.63 (18)
C45—C44—C43—C42	0.1 (3)	C51—C44—C43—C42	−177.03 (18)
C45—C44—C51—C52	163.4 (2)	C24—C17—C16—C15	179.40 (19)
C45—C44—C51—C54	−75.5 (3)	C5—C4—C3—C2	0.2 (3)
C45—C44—C51—C53	45.0 (3)	C20—C15—C16—C17	178.8 (2)
C47—C42—C41—O46	−1.7 (3)	C16—C17—C24—C25	−65.8 (3)
C47—C42—C41—C40	177.80 (17)	C16—C17—C24—C26	54.4 (3)
C47—C42—C43—C44	−178.56 (18)	C16—C17—C24—C27	173.6 (2)
C13—C14—C15—C20	−178.64 (19)	C16—C15—C20—C23	−119.0 (2)
C13—C14—C15—C16	0.3 (3)	C16—C15—C20—C21	−0.2 (3)
C13—C18—C17—C24	−179.34 (19)	C16—C15—C20—C22	119.3 (2)
C13—C18—C17—C16	0.1 (3)	C2—C7—C6—C5	0.6 (3)
C8—C7—C6—C5	−179.70 (19)	C32—C31—C30—C29	−0.4 (4)

Symmetry code: (i) −*x*+1, −*y*+1, −*z*+1.

### N'-[(E)-3,5-Di-tert-butyl-2-hydroxybenzylidene]-2-hydroxybenzohydrazide ethanol quatersolvate Hydrogen-bond geometry (Å, º)

**Table:**

*D*—H···*A*	*D*—H	H···*A*	*D*···*A*	*D*—H···*A*
O46—H46···N38	0.84	1.81	2.554 (2)	147
O19—H19···N11	0.84	1.87	2.618 (2)	148
O28—H28···O36	0.84	1.98	2.696 (2)	142
O28—H28···O55	0.84	2.35	2.962 (7)	130
O1—H1···O9	0.84	1.85	2.579 (2)	144
N37—H37···O9	0.88	2.04	2.905 (2)	168
N10—H10···O36^ii^	0.88	2.14	2.970 (2)	158
C12—H12···O36^ii^	0.95	2.57	3.360 (2)	140
C12—H12···O55^ii^	0.95	2.61	3.433 (7)	145

Symmetry code: (ii) *x*−1, *y*, *z*.
